# Association of Lead Exposure with Survival in Amyotrophic Lateral Sclerosis

**DOI:** 10.1289/ehp.11193

**Published:** 2008-04-02

**Authors:** Freya Kamel, David M. Umbach, Lillian Stallone, Marie Richards, Howard Hu, Dale P. Sandler

**Affiliations:** 1 Epidemiology Branch and; 2 Biostatistics Branch, National Institute of Environmental Health Sciences, National Institutes of Health, Department of Health and Human Services, Research Triangle Park, North Carolina, USA; 3 Social and Scientific Systems, Inc., Durham, North Carolina, USA; 4 Westat, Durham, North Carolina, USA; 5 Department of Environmental Health Sciences, School of Public Health, University of Michigan, Ann Arbor, Michigan, USA

**Keywords:** amyotrophic lateral sclerosis, lead, prognosis, survival

## Abstract

**Background:**

Reasons for the variability in survival among ALS cases are unknown but may include exposure to environmental neurotoxicants.

**Objectives:**

We aimed to determine whether lead exposure, assessed by measuring blood and bone lead levels, is associated with survival in amyotrophic lateral sclerosis (ALS).

**Methods:**

We evaluated the relationship of lead exposure to ALS survival in 110 cases from a case–control study conducted in New England in 1993–1996 that included measurements of blood and bone lead. We retrieved information on date and cause of death through 31 December 2003 from the National Death Index Plus and the Social Security Administration Death Index. We evaluated the relationship of survival to lead exposure using Cox proportional hazard analysis, with adjustment for age, sex, and smoking.

**Results:**

We found mortality data for 100 of 110 cases; 93 of 100 death certificates mentioned ALS. Median survival from diagnosis to death was 28 months. Shorter survival was associated with older age at diagnosis, female sex, bulbar onset, shorter interval between symptom onset and diagnosis, and reduced lung function. Shorter survival from diagnosis to death had a weak inverse association with blood lead (hazard ratio = 0.9; 95% confidence interval, 0.8–1.0) and a stronger inverse association with patella lead (0.5; 0.2–1.0) and tibia lead (0.3; 0.1–0.7); similar results were found for survival from symptom onset to death.

**Conclusions:**

These results suggest that lead exposure is associated with longer survival in ALS cases and, if confirmed, may shed light on mechanisms involved in disease progression.

Amyotrophic lateral sclerosis (ALS) is a progressive neurodegenerative disease affecting the motor neurons of the spinal cord and brain. Typically, the disease is rapidly fatal; most individuals die within 2–3 years of diagnosis, often from respiratory failure (Mitchell and Borasio 2007). However, survival is variable, with some individuals living ≤ 10 years (del Aguila et al. 2003). The reasons for this variability are largely unknown. Factors related to shorter survival include older age, female sex, bulbar onset, and decreased time from symptom onset to diagnosis (del Aguila et al. 2003), but other factors likely play a role.

The etiology of ALS is also not well understood, but the disease is generally considered to be a result of the interplay between genetic and environmental factors (Mitchell and Borasio 2007). Sporadic ALS is not strongly related to superoxide dismutase (*SOD*1) or other genes associated with familial ALS, although variation in these or other genes may increase susceptibility (Kunst 2004; Mitchell and Borasio 2007). No environmental exposure has been convincingly demonstrated to play a role in ALS (Armon 2003), but some evidence implicates smoking (Kamel et al. 1999), pesticides (McGuire et al. 1997), electromagnetic fields (Savitz et al. 1998), and heavy metals (Kamel et al. 2005).

In a previous study (Kamel et al. 2002), we found that ALS risk was associated with both blood and bone levels of lead as well as with occupational lead exposure. Other studies have also reported associations of ALS risk with lead exposure (reviewed by Kamel et al. 2005), and a recent study found a cluster of ALS cases in proximity to an active lead smelter in Missouri (Turabelidze et al. 2008). In the present study we followed cases from our previous study (Kamel et al. 2002) to evaluate the relationship of lead exposure to ALS mortality. Our expectation was that lead exposure would shorten survival; surprisingly, we found that lead exposure was associated with longer survival.

## Materials and Methods

We conducted a case–control study in New England, USA, in 1993–1996 (Kamel et al. 2002). Sequential ALS cases were recruited from two Boston, Massachusetts, ALS clinics; 71% of eligible cases participated in the study (*n* = 110). Diagnosis of ALS by board-certified neurologists specializing in motor neuron disease was based on World Federation of Neurology El Escorial criteria, that is, on the presence of progressive disease with both upper and lower motor neuron signs (Brooks 1994). Cases were required to live in New England, to be mentally competent, and to speak English. About 85% of cases were enrolled in the study within 1 year of diagnosis, and the remainder within 2 years; 46% were enrolled within 1 year of symptom onset, 73% within 2 years, and 86% within 3 years.

Data collection involved an in-person structured interview and measurements of blood and bone lead; study procedures were completed within 1 month of enrollment. The interview collected information on demographics, lifestyle, occupational history, medical history, and occurrence of ALS in first-degree relatives; its main focus was exposure to lead. We based self-reported occupational exposure to lead on the question, “On any of your jobs, were you exposed 10 times or more to lead in any form (fumes, dust, particles)?” We also recorded dates of first ALS diagnosis and first experience of related symptoms in this interview. We extracted information on respiratory function [forced vital capacity (FVC)] from medical records and available only for a subset of cases (*n* = 68). We measured bone lead in tibia and patella using K X-ray fluorescence and blood lead using atomic absorption spectrometry (Kamel et al. 2002). Blood lead measurements were available for 107 cases and bone lead measurements for 104. We collected whole blood as a source of DNA, and genotyped the K59N polymorphism (rs1800435) in the δ-aminolevulinic acid dehydratase (*ALAD*) gene using polymerase chain reaction–restriction fragment length polymorphism (Kamel et al. 2003).

For the present study, we obtained information on date and cause of death by searching the National Death Index (NDI) Plus (National Center for Health Statistics, Hyattsville, MD) through 31 December 2003. We considered five items in identifying matches to NDI Plus data: social security number, birth date (day, month, and year), first name, last name, and sex. Seventy-eight individuals were complete matches, with all five items identical. Nineteen were partial matches: either four items were identical, or three items were identical and no more than four digits of the social security number were different. Four poor matches did not meet these criteria, and we found no NDI matches for nine individuals; for 3 of these 13 individuals, death dates were found by searching the records of the Social Security Administration and death certificates were retrieved. Thus, date and cause of death were available for 100 of 110 cases (91%). Comparing the 10 individuals without mortality data with the 100 with data, the former were slightly younger (median age, 57 vs. 61 years) and more likely to be male (8 of 10 vs. 59 of 100), but blood and bone lead levels in the two groups did not differ. ALS was recorded on the death certificate as either an underlying or a contributing cause of death, for 93 of the 100 cases (93%) for whom we had information on date and cause of death.

We used Cox proportional hazard analysis to estimate hazard ratios (HRs) and 95% confidence intervals (CIs) using SAS, version 9.1 (SAS Institute Inc., Cary, NC). We conducted two parallel analyses using as the dependent variable interval from diagnosis to death or interval from symptom onset to death, both defined based on interview data. The 10 cases without mortality data were assumed to be still living and were censored at the end of follow-up; censored individuals contributed person-time to the denominator up to the time of censoring but no events to the numerator. Sensitivity analyses either censoring these individuals at the date of their interview or excluding them gave results similar to those presented below. We censored 7 individuals whose death certificates made no mention of ALS (5 of whom were exact matches) at their date of death, again allowing them to contribute to the denominator but not the numerator. Results were similar if we assumed that these seven individuals had in fact died of ALS and counted them in the numerator.

We modeled blood and bone lead levels as continuous variables after transformation using log_2_([Pb] + 32), where [Pb] is lead concentration (Kamel et al. 2002). Because age, sex, and smoking may be related to both lead exposure and ALS, all models included age at interview (as a log-transformed continuous variable), sex, and a dichotomous variable for ever having smoked; considering smoking status (never, former, current) at the time of the interview gave similar results. Additional factors considered as potential confounders included education (high school or less vs. greater than high school); body mass index (BMI; dichotomized at the lowest quartile) and physical activity level at the time of the interview (hours of sitting, lying down, or sleeping per day, continuous); site of disease onset (bulbar vs. limb); the interval between symptom onset and diagnosis (dichotomized at the median); history of ALS in first-degree relatives; and respiratory function (FVC, dichotomized at the median), assessed in models excluding individuals with missing data. None of these variables changed the effect estimates for lead by > 10%, so none was included in final models.

We considered potential effect modification by age, sex, or interval between symptom onset and diagnosis using stratified models. Too few individuals had bulbar onset, family history of ALS, or diagnosis > 1 year before enrollment for us to consider these groups separately, but we ran models restricted to individuals without these characteristics.

The study was approved by the institutional review boards of participating institutions, and the participants provided written informed consent.

## Results

The median (range) interval between first diagnosis and death for the 100 cases with death certificate information was 28 (6–121) months; the median interval between symptom onset and death was 40 (9–207) months. Personal characteristics were associated with both the diagnosis-to-death and the symptoms-to-death intervals. Older individuals and women survived for a shorter time, whereas those who had ever smoked, had no more than a high school education, or were in the lowest quartile of BMI lived longer, although all results were imprecise (Table 1). Clinical characteristics were also related to both diagnosis-to-death and symptoms-to-death intervals: cases with a longer interval between symptom onset and diagnosis or a family history of ALS lived longer, whereas those with bulbar onset or reduced FVC survived for a shorter time; again, results were imprecise (Table 1).

Blood and bone lead levels of cases were higher than those of controls in the original case–control study (Kamel et al. 2002). After adjustment for age, sex, and area within New England, mean ± SE blood lead levels were 5.2 ± 0.4 μg/dL for cases and 3.4 ± 0.4 μg/dL for controls; patella lead levels were 20.3 ± 2.1 μg/g for cases and 16.7 ± 2.0 μg/g for controls; and tibia lead levels were 14.9 ± 1.6 μg/g for cases and 11.1 ± 1.6 μg/g for controls. Both blood and bone lead levels were inversely associated with survival after adjustment for age, sex, and smoking. The relationship with blood lead was relatively weak, but both patella and tibia lead were strongly associated with intervals of both diagnosis to death and symptoms to death (Table 2). Further adjustment of lead models for education, BMI, physical activity, bulbar onset, interval between symptom onset and diagnosis, family history of ALS, or FVC did not alter the association of lead levels with survival for either outcome. In models of the diagnosis-to-death interval including any one of these variables, HRs for blood lead were 0.91–0.96, HRs for patella lead were 0.4–0.6, and HRs for tibia lead were 0.1–0.4; results were similar for the symptoms-to-death interval (data not shown). Self-reported occupational lead exposure was also inversely associated with survival, although results were imprecise (Table 2).

We evaluated the relationship of ALS survival to each lead variable after stratification by age, sex, or the interval between symptom onset and diagnosis. None of these variables substantially modified the association of ALS survival with blood or tibia lead, but we observed the association with patella lead primarily in individuals > 60 years of age, in men, and in those with an interval of no more than 8 months between symptom onset and diagnosis (Table 2). Exclusion of individuals with a family history of ALS, bulbar onset, or diagnosis > 1 year before enrollment did not affect results substantially. In models of the diagnosis-to-death interval restricted by any one of these three variables, HRs were as follows: for blood lead, 0.9; for patella lead, 0.3–0.5; and for tibia lead, 0.2–0.4. Results were similar for the symptoms-to-death interval (data not shown).

Blood and bone lead levels were correlated (Spearman *r* = 0.4 for blood and patella, 0.4 for blood and tibia, and 0.5 for patella and tibia), so we considered relationships among effects of the three lead variables by modeling them together. For the diagnosis-to-death interval, HRs (95% CIs) were 0.9 (0.8–1.1) for blood lead, 0.8 (0.3–1.7) for patella lead, and 0.4 (0.2–0.9) for tibia lead in a model including all three variables. For the symptoms-to-death interval, HRs were 0.9 (0.8–1.0) for blood lead, 0.9 (0.4–2.2) for patella lead, and 0.4 (0.2–0.9) for tibia lead.

Blood lead levels were not associated with the *ALAD* K59N polymorphism, but bone lead levels were lower in carriers of the polymorphism (homozygotes plus heterozygotes) compared with wild-type homozygotes (patella, 7.3 vs. 14 μg/g; tibia, 8.6 vs. 14 μg/g). The polymorphism was not associated with either the diagnosis-to-death or the symptoms-to-death interval, nor did it affect the association of any of the three lead variables with survival (data not shown).

## Discussion

In this study, we found that lead exposure was associated with longer survival in ALS cases. Results were similar whether we considered the interval between first diagnosis and death or the interval between symptom onset and death. The strongest association was with tibia lead, although blood and patella lead and self-reported occupational exposure to lead were also related to survival.

This observation contrasts with our previous finding that lead exposure was associated with increased risk of ALS (Kamel et al. 2002), thus suggesting that lead exposure may have different effects on ALS onset and progression. This apparently paradoxical result may have a biological explanation. Recent studies in a mouse model of inherited ALS have suggested that onset is related to motor neuron function, whereas progression is regulated by neuroglia (Boillee et al. 2006). Thus, it is possible that the two processes respond differently to neurotoxic exposures. For example, lead might injure motor neurons but stimulate glial cells to provide trophic support to neurons and delay cell death. Support for this hypothesis comes from a recent study showing that although lead was toxic to motor neurons in monoculture, it increased the trophic activity of astrocyte-conditioned culture medium for motor neurons (Barbeito et al. 2005). The same study found that lead exposure prolonged survival in SOD transgenic mice (Barbeito et al. 2005). Our result is also supported by a previous study of lead exposure and survival that found that the 5-year survival rate was 54% in 11 individuals with high lead exposure (based on self-report) but 16% in 89 individuals with low or no lead exposure (Campbell et al. 1970). Thus, it is possible that lead-related ALS involves a mechanism, still unidentified, that generally leads to slower progression than ALS associated with other etiologies.

Alternatively, it is possible that factors associated with better survival were also associated with higher lead levels in our study population. For example, men have higher lead levels (Brody et al. 1994) and live longer with ALS (del Aguila et al. 2003). However, although survival was associated with blood and patella lead only in men, it was strongly related to tibia lead in both men and women. Confounding by age is unlikely to account for our results, because older individuals have higher lead levels (Brody et al. 1994; Hu et al. 1996; Korrick et al. 2002) but live for a shorter time with ALS (del Aguila et al. 2003); in the present study, lead was inversely associated with survival in both younger and older individuals. We observed associations of lead with survival after adjustment for age, sex, and smoking; further adjustment for education, BMI, or physical activity did not change the results, suggesting that confounding by these characteristics did not account for our findings. Adjustment for or stratification by clinical characteristics, including bulbar onset, diagnostic delay, family history of ALS, or respiratory dysfunction, also did not affect the association of lead with survival.

A third possible explanation for the apparently paradoxical influence of lead on ALS onset and survival is that one of the two associations might be artifactual. Biases of various kinds can arise in studies of factors that potentially influence both disease incidence and disease progression (Glymour 2007). For example, if lead exposure were related not to ALS risk but in fact to slower progression (longer survival), or to a more benign (less symptomatic) form of disease, then lead-exposed cases might be more likely to enroll in the study. In contrast, no such selective pressure would exist for controls, and therefore ALS cases as a group might have higher lead levels than controls, creating the appearance of a positive association with incidence (risk). Factors consistently related to ALS survival in previous studies include age, bulbar onset, short diagnostic delay, and respiratory dysfunction (Czaplinski et al. 2006; del Aguila et al. 2003; Paillisse et al. 2005). Other factors potentially related to survival are sex, diagnostic certainty, score on the ALS Functional Rating Scale or similar instruments, and rate of progression after diagnosis (Chio et al. 2002; Guiloff and Goonetilleke 1995; Millul et al. 2005; Turner et al. 2002). Consistent with these reports, we found that older age, female sex, decreased interval between symptom onset and diagnosis, bulbar onset, and reduced FVC shortened survival.

Previous studies have indicated that diagnosed ALS is usually reported on death certificates, although there is some variation between and even within countries. For individuals with ALS who could be traced, ALS was mentioned on the death certificate for 95% of cases in Sweden (Gunnarsson and Palm 1984), 75% in Italy (Chio et al. 1992), 94% in Scotland (Chancellor et al. 1993), and 86% in England (Dean et al. 1994). One study found that ALS was listed on the death certificate for 67% of cases in northern Italy but only 52% in southern Italy (Ragonese et al. 2004). We were able to identify only one study conducted in the United States, which reported that ALS was mentioned on the death certificate for 72% of cases (Hoffman and Brody 1971). In the present study, we found mortality data for 91% of cases from our original study. The NDI search was conducted 7–10 years after enrollment, so it is possible that some of the others were still living, particularly because cases without mortality data were slightly younger and more likely to be male—characteristics associated with longer survival. Cases without mortality data had lead levels similar to those we traced, reducing concern about bias. ALS was listed on the death certificate for 93% of the traced cases, comparable to rates in Sweden and Scotland. These results suggest that death certificates are a reasonable source of mortality data for ALS.

A major strength of the present study is the availability of biological measures of lead exposure. Blood lead is considered to reflect recent exposure, and bone lead to reflect cumulative exposure, especially lead in cortical bone such as tibia, which has a half-life of decades (Hu et al. 2007). The strong association with tibia lead thus suggests that cumulative rather than recent exposure is related to survival.

Study limitations include the small sample size. However, our estimates of the associations of lead with survival were precise, particularly for tibia lead, indicating that our study had sufficient power to address its hypothesis. We had no information on tracheotomy or other treatments that may have prolonged life in some patients, but such treatment is likely to be independent of lead exposure and thus unlikely to account for its relationship with survival. We also lacked information on disability from the ALS Functional Rating Scale or similar instrument. Because cases for the study were drawn from two tertiary care centers, results may not be generalizable to the population as a whole. However, our study was comparable with others in the length of survival as well as factors affecting survival (Czaplinski et al. 2006; del Aguila et al. 2003; Mitchell and Borasio 2007; Paillisse et al. 2005).

In conclusion, we found that greater lead exposure was associated with longer survival in ALS cases, independent of other personal and clinical characteristics affecting survival or lead exposure. The relationship with tibia lead in particular was strong and consistent, suggesting a role for cumulative lifetime exposure. These results must be interpreted with caution, given the unexpected nature of the finding and the small size of the study, and await replication. If confirmed, these findings may shed light on mechanisms involved in disease progression and could suggest a basis for therapies that prolong survival.

## Figures and Tables

**Table 1:** Relationship of survival of ALS cases to personal and clinical characteristics, New England, 1993–1996.

	Adjusted HR (95% CI)^a^		
Characteristic	Median (range) or no. (%)	Diagnosis to death	Symptoms to death
Age at enrollment (years)	60 (30–79)	2.0 (0.8–4.8)	1.4 (0.6–3.4)
Sex			
Male	67 (61)	1.0 (referent)	1.0 (referent)
Female	43 (39)	1.4 (0.9–2.2)	1.4 (0.9–2.1)
Ever smoked			
No	32 (29)	1.0 (referent)	1.0 (referent)
Yes	78 (71)	0.6 (0.4–1.0)	0.7 (0.5–1.2)
Education			
> High school	72 (65)	1.0 (referent)	1.0 (referent)
≤ High school	38 (35)	0.7 (0.4–1.1)	0.7 (0.4–1.1)
BMI			
> 22.2	83 (75)	1.0 (referent)	1.0 (referent)
≤ 22.2	27 (25)	0.7 (0.4–1.1)	0.7 (0.4–1.2)
Physical activity (hours of inactivity per day)	17 (7–24)	1.0 (1.0–1.1)	1.0 (0.9–1.0)
Bulbar onset			
No	88 (80)	1.0 (referent)	1.0 (referent)
Yes	22 (20)	1.4 (0.8–2.4)	1.3 (0.8–2.3)
Symptom onset to diagnosis (months)			
≤ 8	56 (51)	1.0 (referent)	1.0 (referent)
> 8	54 (49)	0.6 (0.4–1.0)	0.4 (0.2–0.6)
Family history of ALS			
No	102 (93)	1.0 (referent)	1.0 (referent)
Yes	8 (7)	0.5 (0.2–1.2)	0.6 (0.2–1.4)
FVC			
> 2.8 L	34 (50)	1.0 (referent)	1.0 (referent)
≤ 2.8 L	34 (50)	1.8 (0.9–3.6)	1.5 (0.8–3.1)
Missing	42		

a HRs and 95% CIs were calculated with Cox proportional hazard analysis; all models included age, sex, and ever smoked.

**Table 2:** Relationship of survival of ALS cases (*n* = 110) to lead exposure, New England 1993–1996.

		Adjusted HR (95% CI)^a^	
Lead exposure/characteristic	Median (range) or no. (%)	Diagnosis to death	Symptoms to death
Blood lead (μg/dL)^b^			
All	4 (0.5 to 14)	0.9 (0.8 to 1.0)	0.9 (0.8 to 1.0)
Age (years)			
≤ 60	4 (0.5 to 13)	1.1 (0.9 to 1.3)	1.1 (0.9 to 1.3)
> 60	4 (1 to 14)	0.9 (0.8 to 1.0)	0.9 (0.8 to 1.0)
Sex			
Male	4 (1 to 14)	0.9 (0.8 to 1.0)	0.8 (0.7 to 0.9)
Female	4 (0.5 to 13)	1.1 (0.9 to 1.3)	1.1 (0.9 to 1.3)
Symptom onset to diagnosis (months)			
≤ 8	4 (0.5 to 14)	0.9 (0.8 to 1.0)	0.9 (0.8 to 1.0)
> 8	4 (0.5 to 11)	0.9 (0.8 to 1.1)	0.9 (0.8 to 1.1)
Patella lead (μg/g)^b^			
All	15 (0 to 107)	0.5 (0.2 to 1.0)	0.6 (0.3 to 1.2)
Age (years)			
≤ 60	11 (1 to 33)	0.2 (0.1 to 1.2)	0.4 (0.1 to 1.7)
> 60	24 (0 to 107)	0.6 (0.3 to 1.3)	0.6 (0.2 to 1.3)
Sex			
Male	16 (0 to 107)	0.1 (0.0 to 0.3)	0.1 (0.0 to 0.3)
Female	14 (0 to 59)	1.0 (0.3 to 3.3)	1.6 (0.5 to 5.2)
Symptom onset to diagnosis (months)			
≤ 8	18 (0 to 107)	0.2 (0.1 to 0.6)	0.2 (0.1 to 0.6)
> 8	14 (0 to 46)	0.5 (0.2 to 1.5)	0.5 (0.2 to 1.5)
Tibia lead (μg/g)^b^			
All	13 (−6 to 61)	0.3 (0.1 to 0.7)	0.3 (0.2 to 0.7)
Age (years)			
≤ 60	11 (0 to 29)	0.3 (0.1 to 1.2)	0.3 (0.1 to 1.4)
> 60	20 (−6 to 61)	0.2 (0.1 to 0.7)	0.2 (0.1 to 0.6)
Sex			
Male	13 (0 to 61)	0.2 (0.1 to 0.8)	0.2 (0.1 to 0.8)
Female	14 (−6 to 47)	0.4 (0.1 to 1.1)	0.4 (0.2 to 1.4)
Symptom onset to diagnosis (months)			
≤ 8	13 (0 to 61)	0.3 (0.1 to 0.9)	0.3 (0.1 to 1.0)
> 8	14 (−6 to 47)	0.3 (0.1 to 0.7)	0.2 (0.1 to 0.6)
Occupational lead exposure			
No	67 (66)	1.0 (referent)	1.0 (referent)
Yes	35 (34)	0.7 (0.5 to 1.2)	0.7 (0.4 to 1.2)
Missing	8		

a HRs and 95% CIs were calculated with Cox proportional hazard analysis; models included age, sex, and ever smoked, except for sex-stratified models, which included age and ever smoked.

b HRs were calculated for each μg/dL blood lead and for each doubling of bone lead, as described in the “Materials and Methods.”
